# Predicting antibiotic resistance genes and bacterial phenotypes based on protein language models

**DOI:** 10.3389/fmicb.2025.1628952

**Published:** 2025-09-08

**Authors:** Boqian Wang, Renjie Meng, Zhong Li, Mingda Hu, Xin Wang, Yunxiang Zhao, Zili Chai, Yuan Jin, Junjie Yue, Wei Chen, Hongguang Ren

**Affiliations:** ^1^Laboratory of Advanced Biotechnology, Beijing Institute of Biotechnology, Beijing, China; ^2^School of Computer, National University of Defense Technology, Changsha, China; ^3^Department of Stomatology, Hainan Hospital of Chinese PLA General Hospital, Sanya, China

**Keywords:** ARGs, phenotypes, protein language models, deep learning, LSTM

## Abstract

**Introduction:**

Antibiotic resistance is emerging as a critical global public health threat. The precise prediction of bacterial antibiotic resistance genes (ARGs) and phenotypes is essential to understand resistance mechanisms and guide clinical antibiotic use. Although high-throughput DNA sequencing provides a foundation for identification, current methods lack precision and often require manual intervention.

**Methods:**

We developed a novel deep learning model for ARG prediction by integrating bacterial protein sequences using two protein language models, ProtBert-BFD and ESM-1b. The model further employs data augmentation techniques and Long Short-Term Memory (LSTM) networks to enhance feature extraction and classification performance.

**Results:**

The proposed model demonstrated superior performance compared to existing methods, achieving higher accuracy, precision, recall, and F1-score. It significantly reduced both false negative and false positive predictions in identifying ARGs, providing a robust computational tool for reliable gene-level resistance detection. Moreover, the model was successfully applied to predict bacterial resistance phenotypes, demonstrating its potential for clinical applicability.

**Discussion:**

This study presents an accurate and automated approach for predicting antibiotic resistance genes and phenotypes, reducing the need for manual verification. The model offers a powerful technical tool that can support clinical decision-making and guide antibiotic use, thereby addressing an urgent need in the fight against antimicrobial resistance.

## 1 Introduction

Bacterial antibiotic resistance transmission has become one of the greatest threats to global public health, with an estimated 700,000 deaths worldwide attributed to bacterial resistance, and this number is expected to rise to 10 million by 2050 (Sunuwar and Azad, 2021; Lázár and Kishony, 2019). Antibiotic Resistance Genes (ARGs) can be transmitted between different strains through various mediums such as food, water, animals, and humans, with hospital environments particularly facilitating the spread of resistant phenotypes and reducing the efficacy of antibiotic treatments (Karkman et al., 2018; Wang et al., 2018). Therefore, accurately identifying resistance genes and predicting strain resistance phenotypes is crucial for guiding clinical medication.

The advent of high-throughput DNA sequencing technology now provides a powerful tool for profiling the entire DNA complement, including ARGs, which encode proteins that confer resistance to antibiotics (Arango-Argoty et al., 2018). Focusing on DNA/protein sequences, bioinformatics is widely applied in the identification and analysis of resistance genes. Traditional identification methods are based on the computational principle of comparison of the ARGs database, using programs such as BLAST, Bowtie, or DIAMOND with a preset similarity cutoff and alignment length requirement (Boolchandani et al., 2019; Lakin et al., 2017; Li and Durbin, 2009). However, the false negative rate can be very high, that is a large number of actual ARGs will be predicted as non-ARGs by the best hit approaches above (Arango-Argoty et al., 2018). At the same time, the high sequence similarity between some non-resistant and resistant genes may also lead to false-positive predictions (Mathys et al., 2014; Fitzgibbon et al., 2021).

Comparatively, the AI-based algorithm for ARGs prediction demonstrated superior predictive performance, which could effectively reduce both false-negative and false-positive prediction outcomes simultaneously (Sunuwar and Azad, 2021; Alley et al., 2019; Li et al., 2018; Riesselman et al., 2018; Su et al., 2019; Sakagianni et al., 2023; Kim et al., 2022). For example, DeepARG (Arango-Argoty et al., 2018) effectively identifies ARGs by comparing experimental sample data with known sequences using a multilayer perceptron model. Additionally, deep learning methods like HMD-ARG (Li et al., 2021) have successfully distinguished between various resistance gene antibiotic group categories. These methods uniformly employ either conventional or deep learning models, which not only exhibit poor interpretability but also yield predictions constrained by training data, resulting in limited scalability.

To solve the problems above, we designed a novel ARGs prediction model by integrating pretrained protein language models for feature encoding and long short-term memory (LSTM) networks with multi-head (MH) attention mechanisms for feature extraction (Elnaggar et al., 2021; Al-Deen et al., 2021; Yu et al., 2019; Rives et al., 2021). Since this model is primarily based on large-scale pretrained protein encoding processes, it can enhance biological interpretability from the perspective of protein linguistics while simultaneously improving scalability for predicting diverse bacterial proteins. Finally, a comparison with traditional nucleotide-based (best hit) and emerging AI-based ARGs identification methods shows that our model outperforms these methods in various metrics such as accuracy, precision, recall, and F1-score, which means a significantly reduction of both false-negative and false-positive prediction rates across different microbial communities.

## 2 Materials and methods

### 2.1 Overall framework

**Figure 1:**
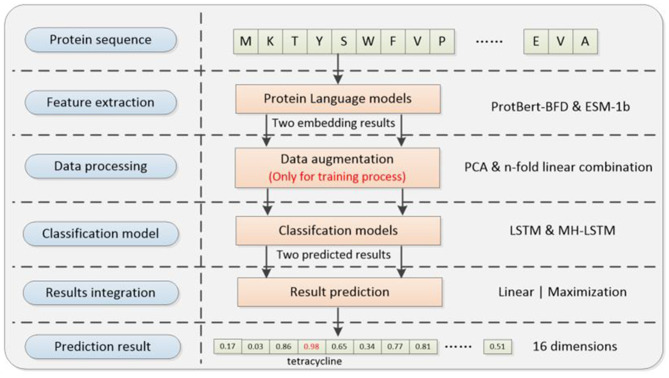
Overall framework of the system architecture. (1) Feature extraction: this step utilizes two protein language models, ProtBert-BFD and ESM-1b, which focus on different structural information of proteins to construct two sets of embedding feature datasets. (2) Data processing: this step utilizes a cross-referencing data augmentation method based on the ProtBert-BFD and ESM-1b embedding results to address the issue of data imbalance (only for training process). (3) Classification model: by exploring different model structure combinations, it assesses the adaptability of various models in capturing different feature vectors, given their varied focus. (4) Result integration: it includes two different ensemble learning strategies to integrate results from multiple models, enhancing the overall generalization performance and predictive effectiveness of the system.

The deep learning framework (Figure 1) proposed in this paper consists of four main modules, which are separately feature extraction module, data processing module, classification model and result integration module. The relevant codes can be found on GitHub: https://github.com/wr-sky/ARGs/tree/main/Code.

Firstly, we utilize two different protein language models [ProtBert-BFD (Elnaggar et al., 2021) & ESM-1b (Rives et al., 2021)] to extract features from proteins sequences, which can facilitate both data augmentation and prediction accuracy compared with the single-language model. Secondly, by cross-referencing two protein language models, we designed a novel data augmentation method to enhance less prevalent ARGs examples during training process, which makes the training set more balanced. Thirdly, we used two semantic-based encoding models (LSTM & MH-LSTM) to classify the embedding results separately from ProtBert-BFD and ESM-1b. Finally, our framework provides a 16-dimension vector by integrating two classification results above. The position with the maximal value will be chosen and its corresponding ARGs type is the final prediction result.

### 2.2 Protein sequence

To ensure the authority and comparability of the data, this study primarily uses data from DeepARG and HMD-ARG as the basic ARGs dataset (Arango-Argoty et al., 2018; Li et al., 2021). Besides, 2,000 non-resistant genes reported in HyperVR (Ji et al., 2023) were included for related experiments. Protein sequences in three datasets (Table 1) are compared with *blastp*, removing completely identical sequences (identity = 100% & coverage = 100%). For ease of reference and result reproduction, detailed information of the protein sequence in Table 1 are uploaded to GihHub: https://github.com/wr-sky/ARGs/tree/main/Data.

**Table 1:** Training and testing dataset composition.

**Antibiotic group**	**Tag**	**Number (HDM-ARG-DB)**	**Number (DeepARG-DB)**	**Others**
Macrolide-lincosamide-streptogramin	0	1,287	1,106	Kasugamycin
Multidrug	1	1,338	1,091	Peptide
Others	2	260	207	Fosmidomycin
Tetracycline	3	381	266	Tetracenomycin
Quinolone	4	297	132	Fusidic_acid
Aminoglycoside	5	1,249	869	Mupirocin
Bacitracin	6	4,219	4,206	Triclosan
Beta_lactam	7	5,921	5,195	Thiostrepton
Fosfomycin	8	351	292	Tunicamycin
Glycopeptide	9	316	223	Qa_compound
Chloramphenicol	10	488	470	Streptothricin
Rifampin	11	68	26	Puromycin
Sulfonamide	12	91	20	Elfamycin
Trimethoprim	13	122	82	Peptide
Polymyxin	14	935	897	Bleomycin
Total_1 (Low-quality data removed)	-	17,282	14,957	Aminocoumarin
Non-ARGs	15	2,000 (HyperVR)		Acriflavin
Total_2 (Redundant data removed)	-	20,981		Multidrug-mutation

The dataset includes data from DeepARG, HMD-ARG and HyperVR. DeepARG data mainly comes from three public resistance gene databases: CARD, ARDB, and UNIPROT; The HMD-ARG dataset is sourced from multiple public databases including CARD, AMRFinder, ResFinder, ARG-ANNOT, DeepARG, MEGARes, and Resfams. The 2,000 non-resistant genes reported by HyperVR come from the UNIPROT database. Additionally, all samples were categorized into 16 antibiotic groups and resistance genes with fewer samples were integrated into “other” group following the standard in HMD-ARG. Small-size data are marked by red font.

All ARGs in Table 1 are categorized into 16 groups. Aside from the ARGs categorized as “other”, some resistance gene groups (marked in black) are more abundant, particularly those associated with bacitracin and beta-lactam resistance. In contrast, the remaining resistance gene groups (marked in red) are less prevalent. Each protein sequence, including the resistance or non-resistance genes, will be taken as initial input for our proposed framework.

### 2.3 Feature extraction

ARGs often contribute to bacterial metabolism through specific structures (both 2D and 3D) of the proteins they encode, resulting in resistance by degrading, obstructing, or expelling antibiotics (Darby et al., 2023; Kakoullis et al., 2021). We have analyzed existing protein language models and their characteristics (Supplementary material) (Elnaggar et al., 2021; Lin et al., 2023; Rao et al., 2020; Meier et al., 2021; Hsu et al., 2022). Based on the specifics of this study, we selected two pre-trained protein language models as upstream feature extractors to embed information carried by protein sequences. The ProtBert-BFD model extracts embedding vectors that capture key information from protein sequences and is also used in downstream tasks such as secondary structure prediction (Elnaggar et al., 2021). The ESM-1b model, through logistic regression and linear projection, encodes embedding features containing the secondary and tertiary structural information of protein sequences (Rives et al., 2021). Thus, this step employs these two models as feature extraction methods, embedding the sequence and structural features of the target proteins from different dimensions.

Both models take the protein sequence as input. ProtBert-BFD encodes each amino acid as a 30-dimensional vector. Each protein sequence is encoded into a 30,720-dimensional vector by padding 0 or vector truncation (1,024 amino acids). Similarly, ESM-1b encodes each amino acid as a 1,280-dimensional vector and each protein sequence is encoded into a 1,310,720-dimensional vector (1,024 amino acids).

### 2.4 Data augmentation

**Figure 2:**
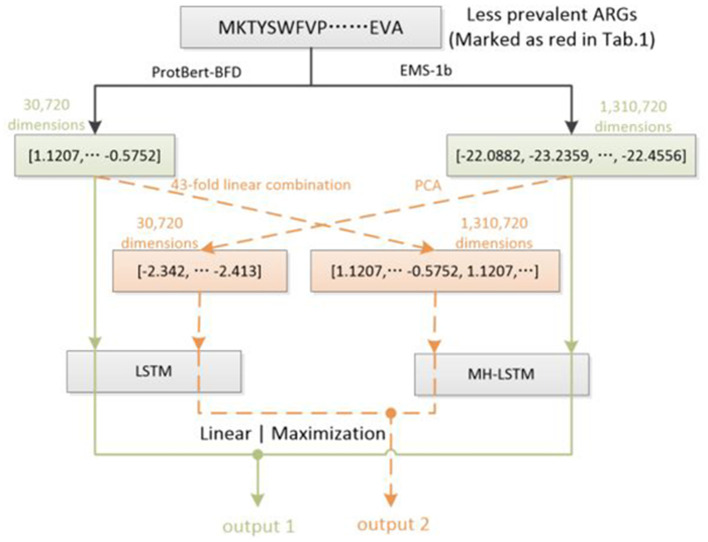
Dataset augmentation based on ProtBert-BFD and ESM-1b embedding results. For ESM-1b, the embedding results of each amino acid (1,280 dimensions) will be decreased to 32 dimensions by PCA. Each embedding results will be concatenated in order to represent a protein sequence (overall 32,768 dimensions) and truncated at the end according to the ProtBert-BFD's embedding results (30,720 dimensions). For ProtBert-BFD, the embedding results of the whole protein sequence (30,720 dimensions) will be extended using a 43-fold linear concatenation to 1,320,860 dimensions, which will be further truncated at the end according to the EMS-1b's embedding results (1,310,720 dimensions). By this way, each example in less prevalent ARGs will be utilized twice during training process, which can potentially double the training set of the corresponding ARGs.

In Natural Language Processing (NLP) tasks, data augmentation for small datasets is a crucial strategy to enhance model performance and generalization (Chen et al., 2023). However, due to differences between resistance gene protein sequences and natural language features, traditional NLP data augmentation strategies cannot be directly applied to this task. Therefore, For the first time, we designed a new data augmentation method for the limited antibiotic resistance data (Figure 2). This method exponentially increased the limited amount of resistant gene data (marked as red in Table 1), making the input data for each type of resistant gene more balanced.

We utilize Principal Component Analysis (PCA) (Hasan and Abdulazeez, 2021) to decrease ESM-1b's embedding results of each amino acid from 1,280 dimensions to 32 dimensions. The overall dimension of a protein sequence's (1,024 amino acids) embedding results is 32,768, which will further be truncated to 30,720 dimensions. For the embedding results of ProtBert-BFD, we use a 43-fold linear concatenation to directly extend the feature vector of the entire protein to 1,320,860 dimensions, which is also truncated to 1,310,720 dimensions. For each input, the green line forms one training process, and the orange line forms another training process, which can double the examples in less prevalent ARGs (red groups in Table 1).

The overall test results, which will be illustrated in the “Results” section below, provide evidence to support the feasibility of such a transformation between the two embedding spaces.

### 2.5 Classification model

**Figure 3:**
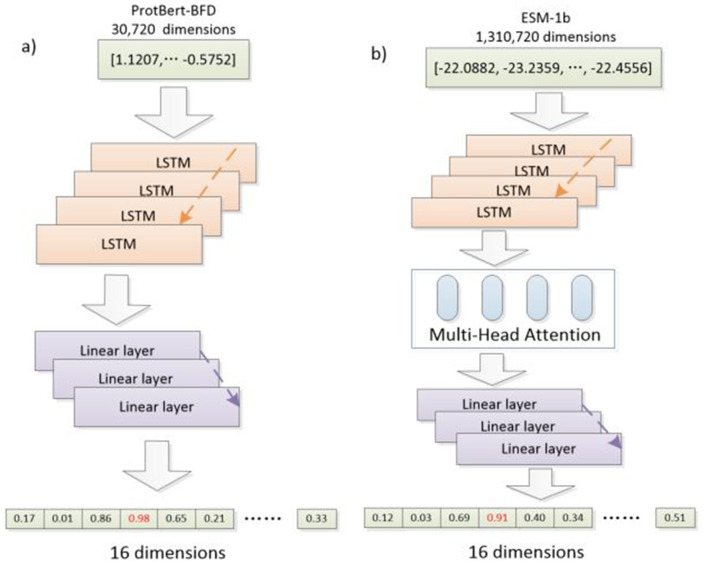
The architectures of classification models. **(a)** LSTM: A LSTM model for classification is utilized for the relatively lower-dimensional encoded data from ProtBert-BFD and its augmented data. **(b)** MH-LSTM: A classification model that integrates LSTM with MH is utilized for the high-dimensional encoded data from ESM-1b and its augmented data.

LSTM is well-suited for handling long-range dependencies in language data and can effectively capture contextual information in sequential data, making it particularly suitable for processing the temporal nature of linguistic data (Yu et al., 2019). On the other hand, MH-LSTM introduces a multi-head mechanism that allows for the parallel processing of multiple types of contextual information, further enhancing the model's ability to understand the complex syntax, semantics, and ambiguity in language. Therefore, both LSTM and MH-LSTM are ideal choices for processing linguistically encoded data, as they are better at capturing the complex dependencies and multiple layers of contextual information in language, ultimately improving the model's performance and expressive power. In this paper, we employed Multi-Head Attention LSTM (MH-LSTM) and LSTM to extract effective information while reducing the dimensionality of the protein embedding vector (Figure 3). By mixing different models, MH-LSTM can fully extract features from high-dimensional embedding results (ESM-1b), while LSTM can avoid over-abstraction of relatively low-dimensional embedding results (ProtBert-BFD).

The input feature size is 30,720 dimensions for LSTM and 1,310,720 dimensions for MH-LSTM. Both models have hidden layers and output layers of size 512. The final classification is performed by a linear layer with GELU activation, which takes input sizes of 512, 1,024, and 2,048 across three layers (Table 2). These architecture parameters were determined through experimentation and preliminary trials. The output of the linear layer is a 16-dimensional vector, with each dimension representing a group of ARGs (Table 1).

**Table 2:** The optimal parameters for each structure.

**System structure**	**Classification model**	**Number of attention heads**	**Deepth of LSTM**	**Deepth of linear layer**	**Structure of linear layer**	**Dropout**	**Loss**	**Learning rate**
LSTM_MH-LSTM_LINEAR	LSTM	-	3	3	512 × 1,024	0.256	0.5	0
					1,024 × 2,048	0.456		
					2,048 × 16	0.365		
	MH-LSTM	6	4	3	512 × 1,024	0.256	0.5	0
					1,024 × 2,048	0.456		
					2,048 × 16	0.365		
LSTM_MH-LSTM_MAX	LSTM	-	3	3	512 × 1,024	0.256	0.6	0
					1,024 × 2,048	0.456		
					2,048 × 16	0.365		
	MH-LSTM	4	5	3	512 × 1,024	0.256	0.4	0
					1,024 × 2,048	0.456		
					2,048 × 16	0.365		

Different structures of each system (LSTM_MH-LSTM_LINEAR & LSTM_MH-LSTM_MAX) were tested to find out the optimal parameters suitable for ProtBert-BFD and ESM-1b embedding results. The parameters include number of attention heads (only for MH-LSTM), depth of LSTM, depth of linear layer (fixed), structure of linear layer (based on input and output dimensions), dropout (fixed), loss rate, and learning rates (fixed). A deeper network structure in MH-LSTM model is required compared with LSTM and more heads is necessitated for linear integration algorithm compared with the max integration algorithm.

### 2.6 Result integration

**Figure 4:**
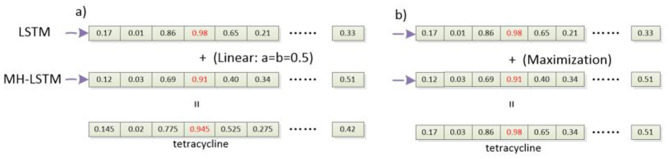
The process of integrating classification results. **(a)** Linear integration: linear integration involves process to weight and sum the prediction probabilities of the two models. **(b)** Probability maximization integration: probability maximization integration, on the other hand, compares the probability values of the predicted labels from both models and the label associated with the higher probability is chosen as the final prediction.

By using ensemble learning, combining the predictions of two classification models can effectively reduce overfitting (Ying, 2019). At the same time, it increases the diversity and robustness of the model, thereby potentially improving prediction accuracy. In our proposed framework, we process the results of the two models using linear integration or probability maximization integration methods (Figure 4).

Especially, for linear integration (Figure 4a), the probability values output by the two models are linearly combined, as described by Equations 1, 2.


(1) $$\begin{array}{ccc} logits & = & a\;\times logits\;1+b\;\times logits\;2 \end{array}$$

(2) $$\begin{array}{c} y\text{\_}hat\;=argmax\left(logits\right) \end{array}$$

In this context, *logits*1 represents the probability vector output by the LSTM network, and *logits*2 represents the probability vector output by the MH-LSTM. *a* and *b* are constant coefficients, and their sum equals 1. Both a and b are initialized to 0.5 and dynamically adjusted during the training process. The *argmax*() function returns the index *y*_*hat* corresponding to the maximum probability in the *logits*, which indicates the final predicted label.

For probability maximization integration (Figure 4b), the probabilities corresponding to their predicted labels are compared, and the label associated with the higher probability is selected as the final prediction, as described by Equations 3, 4.


(3) $$\begin{array}{c} \max\text{\_}prob\text{\_}1,\max\text{\_}index\text{\_}1=\;max\left(logits\text{\_}1\right) \end{array}$$

(4) $$\begin{array}{c} \max\text{\_}prob\text{\_}2,\max\text{\_}index\text{\_}2=\;max\left(logits\text{\_}2\right) \end{array}$$

max_*prob*_1 and max_*prob*_2 represent the maximum probability values in the probability vectors output by the two models. max_*index*_1 and max_*index*_2 indicate the predicted label types corresponding to these maximum probability values. The *max*() function computes the maximum value and its index in the probability vectors. Then, the sizes of max_*prob*_1 and max_*prob*_2 are compared, and the label associated with the larger probability is chosen as the final predicted type.

Although appropriately increasing the number of models in ensemble learning can usually further improve prediction performance, it is also necessary to consider computational resources and effective fusion methods. Taking these factors into account, we adopt two models (ProtBert-BFD with LSTM & ESM-1b with MH-LSTM) in ensemble learning.

### 2.7 Training and testing

**Figure 5:**
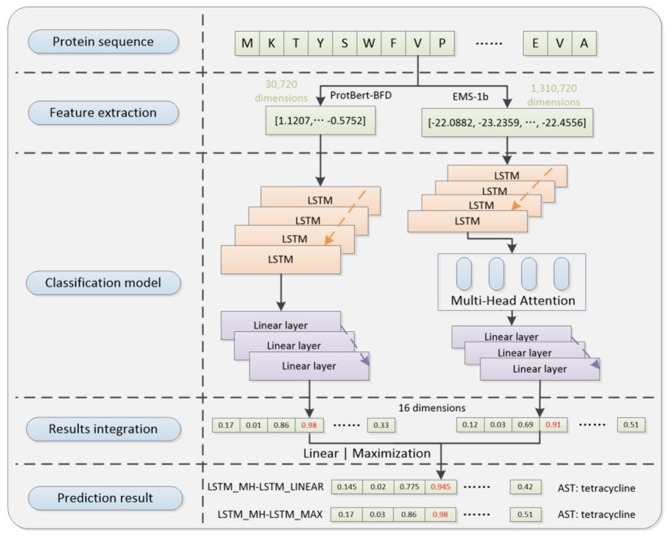
The diagram of LSTM_MH-LSTM_LINEAR and LSTM_MH-LSTM_Max structure in validation and test processes. It includes pre-trained ProtBert-BFD and ESM-1b protein language embedding models in the feature extraction step, LSTM and MH-LSTM language-analysis models (with linear layers) in the classification step, and linear or maximization algorithm in the results integration step.

Based on the aforementioned structures, we developed two overall architectures of the prediction model: Linear-integration-based architecture (LSTM_MH-LSTM_LINEAR) and Probability-maximization-based architecture (LSTM_MH-LSTM_MAX). We constructed datasets for training, validation, and test purposes. The training process includes data processing step, and the other two processes exclude the step (Figure 5).

We used the *train_test_split* function from the *scikit-learn* machine learning toolkit in *Python* to partition the data into training, validation, and test sets with a ratio of 0.6/0.2/0.2. We also selected Adam optimizer with a learning rate of 2e-4 for network training. To avoid overfitting, the optimizer includes a training termination mechanism: if the accuracy does not improve after 30 iterations, the training process will be terminated early, and the model weights will be saved. The final experimental results are obtained by testing the model on the test set. Performance evaluation is conducted using four metrics: accuracy, precision, recall, and F1 score, which provide a comprehensive assessment of the model's performance. Their mathematical expressions are shown in Equations 5–8, respectively.


(5) $$\begin{array}{c} Accuracy=\;\frac{TP+TN}{TP+TN+FP+FN} \end{array}$$

(6) $$\begin{array}{c} Precision=\;\frac{TP}{TP+FP} \end{array}$$

(7) $$\begin{array}{c} Recall=\;\frac{TP}{TP+FN} \end{array}$$

(8) $$\begin{array}{c} F1=\;2\times \frac{Precision\;\times \;Recall}{Precision+\;Recall} \end{array}$$

In this context:

- TP (True Positive): The sample is positive, and the prediction is also positive.
- FP (False Positive): The sample is negative, but the prediction is positive.
- TN (True Negative): The sample is negative, and the prediction is also negative.
- FN (False Negative): The sample is positive, but the prediction is negative.

Precision represents the proportion of samples predicted as positive that are actually positive. Recall represents the proportion of actual positive samples that are correctly predicted as positive. The F1 Score considers both precision and recall, providing a comprehensive measure of the model's performance. These four metrics collectively account for both false-negative and false-positive scenarios. When all metrics approach 1, it indicates superior model performance with minimized misclassification rates for both negative and positive samples.

### 2.8 AMR phenotype prediction

Predicting the presence of ARGs fundamentally indicates whether a bacterial strain has the potential to develop a resistant phenotype. Comparatively, direct prediction of phenotypic resistance can more effectively guide clinical antibiotic selection for bacterial infections. However, defining the antimicrobial resistance (AMR) phenotype of a target bacterial strain solely based on the aggregate features of all its resistance genes would lead to substantially elevated false-positive prediction rates. Therefore, in this subsection, we further try to adapt the architecture of our previously proposed model to predict the whole-bacterial antimicrobial resistance (AMR) phenotypes.

In critical care, combination antibiotic therapy is often employed to achieve the most rapid therapeutic effect. Therefore, false-positive predictions should be rigorously minimized, as they may lead to the avoidance of first-line antibiotics that would have been most effective. In contrast, false-negative predictions in the context of multi-antibiotic regimens typically have less detrimental impact on overall therapeutic efficacy. As traditional best-hit approach has a low false-positive rate (Arango-Argoty et al., 2018), we incorporated this approach [CARD with blast (Alcock et al., 2023)] into our model as a whole-genome screening tool at the bacterial species level. The incorporation of CARD serves dual purposes: primarily filter out both negative and false-positive genes to reduce false-positive AMR predictions, while concurrently reducing computational load for downstream AI networks to accelerate the entire prediction pipeline.

**Figure 6:**
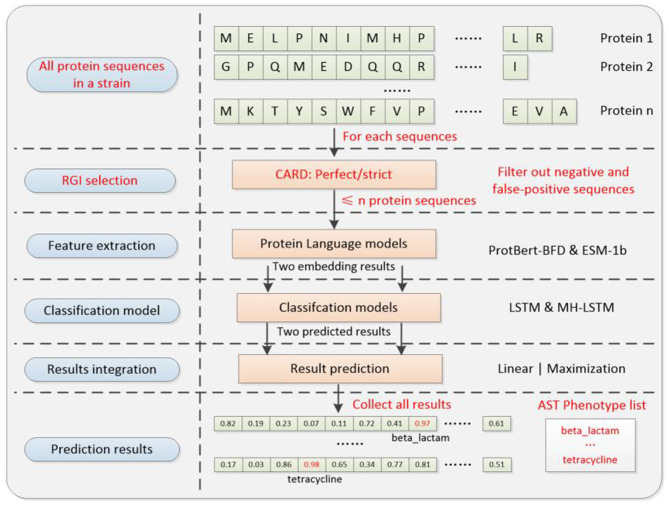
Transfer application system architecture diagram. The well-trained parameter in the classification and results integration models will be directly utilized for transfer application. Compared with the ARGs classification model, it additionally includes an RGI selection step, which could pre-filter most noise genes (negative and false-positive), to improve the overall prediction accuracy of the system. The AST phenotype prediction results are the collection of each protein sequence's ARG prediction result.

The training and validation processes are omitted and only the test process is committed. The experimental steps (Figure 6) are as follows.

1) Data collection: we processed the proteome of each bacterial strain as a complete testing procedure. Each individual protein sequence from the strain was sequentially fed as input to our prediction system and the phenotype prediction result for the strain is the sum of the results for each protein.
2) Protein sequence screening: each protein sequence will be primarily screened by CARD RGI algorithm (v3.1) before the prediction process. RGI screening standards are categorized into Strict and Perfect. For downstream analysis, we retained only proteins receiving positive predictions while filtering out all negative predictions, thereby substantially minimizing false-positive identifications. The relevant codes can be found on GitHub: https://github.com/wr-sky/ARGs/tree/main/Code/3_CARD.
3) Feature Extraction and Prediction (Similar to ARGs classification): for each protein sequence in a single strain, embedding results are extracted using ProtBert-BFD and ESM-1b. These results are then processed by the LSTM (suitable for lower-dimensional ProtBert-BFD data) and MH-LSTM (suitable for higher-dimensional ESM-1b data) with the best model parameters to realize transfer application prediction.

## 3 Results

### 3.1 LSTM and MH-LSTM performance testing

**Figure 7:**
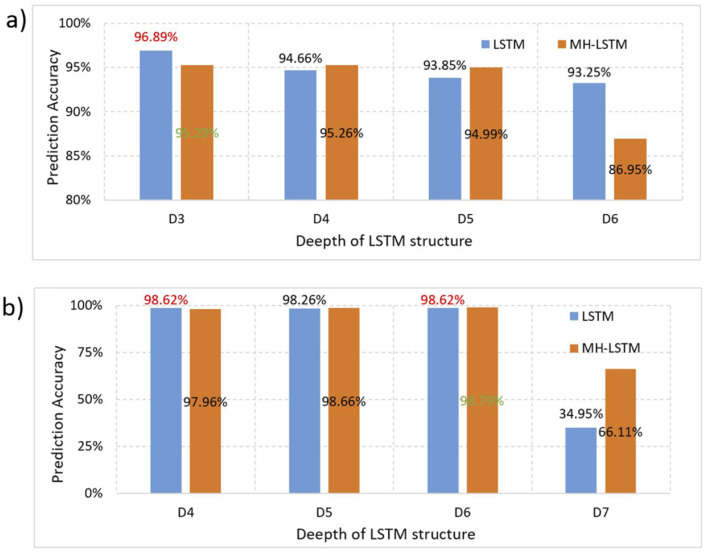
Performance test results separately based on the ProtBert-BFD and ESM-1b encoding results. **(a)** ProtBert-BFD: LSTM and MH-LSTM with depths ranging from 3 to 6 layers (D3-D6) were constructed to test their prediction accuracy. Both LSTM and MH-LSTM achieve the highest performance with 3 layers network structure and the LSTM yielded a higher accuracy compared to the MH-LSTM. **(b)** ESM-1b: Since ESM-1b results in a higher embedding dimensions, LSTM and MH-LSTM with deeper network ranging from 4 to 7 layer (D4–D7) were tested. The results show that multi-layer MH-LSTM networks perform better than LSTM and deeper network (D6) achieved higher performance in this case.

Based on the original data from DeepARG-DB, we test the LSTM and MH-LSTM performance on the ProtBert-BFD (Figure 7a) and ESM-1b (Figure 7b) embedding results respectively. For the ProtBert-BFD, LSTM with 3 layers network structure achieved the best performance with a clear advantage compared with other layers and MH-LSTM structures. This is likely because the low-dimensional data (30-dimensional ProtBert-BFD encoding per amino acid) is more suitable for small-scale, shallow networks, while the multi-head attention mechanism in MH-LSTM may lose some critical information. The embedding results of ESM-1b, which encodes each amino acid into 1,280 dimensions, is significantly higher than ProtBert-BFD's results. In this case, MH-LSTM with 6 layers network structure achieved the best performance. This improvement is due to the increased overall data dimensionality, which favors deep MH-LSTM networks for effective key data abstraction and extraction, while reducing interference from noisy information. In conclusion, proteins encoded by ProtBert-BFD are more suitable for LSTM structures with fewer layers (D3), while proteins encoded by ESM-1b is better suited for MH-LSTM structures with relatively more layers (D4~D6). However, an excessive number of LSTM layers (D7 as an example) increases the model's parameter count, leading to overfitting, which in turn causes a significant decline in test performance.

### 3.2 Experimental test for ARGs prediction

**Figure 8:**
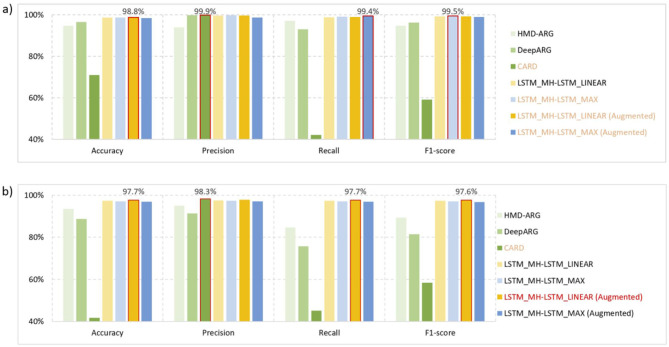
Performance test results. **(a)** ARGs identification: the task of ARGs identification is to distinguish resistant and non-resistant genes from all target genes. Comparatively, our proposed methods (with or without data augmentation) can always deliver satisfactory performance under four evaluation metrics. While CARD achieve slightly higher precision, it performs worst in the other three evaluation metrics. **(b)** ARGs classification: the task of ARGs classification is to classify the 16 different drug resistance gene strategies. The LSTM_MH-LSTM_LINEAR system architecture, based on augmented data, achieved the best results in terms of accuracy, recall, and F1-score. Regarding the precision metric, it is only slightly lower than the CARD method.

Based on the overall dataset (Table 1), we evaluated the prediction performance of different architectures under the optimal structures (Table 2). The optimal structure is determined through repeated experimental iteration with different structural combinations, which also follows the conclusion above. The evaluation was carried out using four metrics: accuracy, precision, recall, and F1-score. We compared our proposed methods with related works, including traditional sequence alignment methods like CARD and machine learning methods such as HMD-ARG and DeepARG. By applying these methods to ARGs identification (Figure 8a) and ARGs classification (Figure 8b) tasks, our proposed model consistently achieved superior performance almost across all evaluation metrics. The only exception is the CARD method under “perfect” criteria, the higher precision of which is due to its more lenient criteria for identifying resistant genes. This kind of criteria will result in a lower false-positive rate but a higher false-negative rate (Arango-Argoty et al., 2018), and consequently, performs the worst under the other three evaluation metrics. Besides, the framework including the data augmentation process usually provides better results compared with the framework without data augmentation technique.

### 3.3 AMR prediction results

We manually screened 262 bacterial strains from NCBI which include both complete antibiotic susceptibility test (AST) results and whole-genome sequences. The strains involve *S.enterica, E.coli, K.pneumoniae, C.freundii, S.marcescens*, and etc. Each strain was annotated based on their AST results and our proposed 16 resistance labels (Table 1). For ease of replication studies, details of each stain can be found on GitHub: https://github.com/wr-sky/ARGs/blob/main/Data/AST_NCBI_id.txt.

In our application pipeline, we tested both “perfect” and “strict” screening standards (CARD RGI) as pre-screening tools for each protein sequence. Basically, the “strict” standard is relatively more lenient than the “perfect” standard, allowing us to optimally preserve high-fidelity resistance genes.

**Figure 9:**
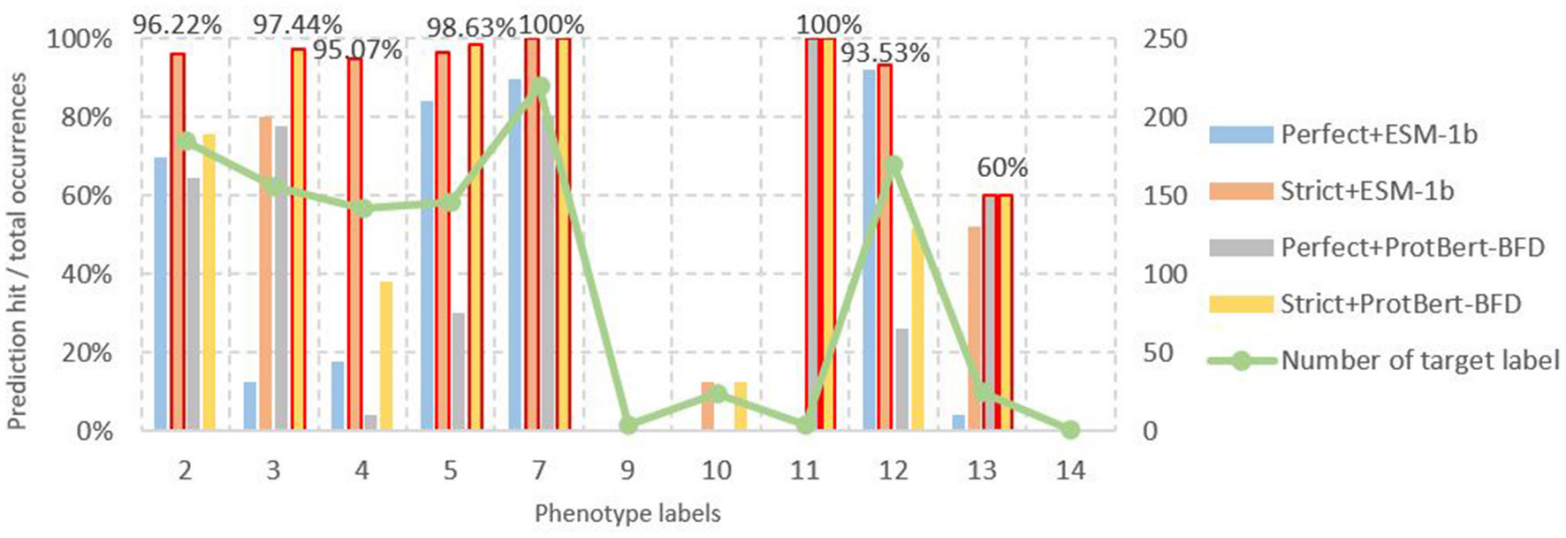
Number of different phenotype labels and their corresponding prediction accuracy results. In the results of ESM-1b based on MH-LSTM architecture, labels 2 (other), 4 (Quinolone), 5 (Aminoglycoside), 7 (Beta-lactam), and 12 (Sulfonamide) all achieve prediction accuracies above 95%, with label 7 reaching 100% precision. For the ProtBert-BFD based on MH-LSTM architecture, labels 3 (Tetracycline), 5 (Aminoglycoside), 7 (Beta-lactam), and 11 (Rifampin) achieve prediction accuracies above 95%, with labels 7 and 11 both reaching 100% precision. Annotations for the remaining groups, which are separately 0, 1, 6, 8, and 15, were missing among the 262 strains from NCBI.

To comprehensively demonstrate predictive performance, we separately quantified model outputs encoded by ESM-1b (right panel of Figure 5 with MH-LSTM) and ProtBert-BFD (left panel of Figure 5 with LSTM). From the perspective of screening criteria, the test results show that datasets filtered by the RGI strict criteria achieve higher prediction accuracy (Equation 5) both for the ESM-1b and ProtBert-BFD embedding results (Figure 9). Compared to the “perfect” standard, the “strict” criteria effectively eliminate both negative and false-positive genes, reducing false-positive (FP) prediction probability and consequently enhancing overall prediction accuracy. From the perspective of embedding models, the ESM-1b model demonstrated superior prediction accuracy for label 2 (other), 4 (quinolone), and 12 (sulfonamide), whereas ProtBert-BFD achieved higher precision for label 3 (tetracycline) and 5 (aminoglycoside). Notably, both models attained 100% accuracy in predicting label 7 (β-lactam) and 11 (rifampin). When combining these two models' results, the prediction accuracy can theoretically exceed 90%, with peak performance reaching 100% for specific antibiotic classes. Overall, our model demonstrates exceptional performance in predicting specific resistance phenotypes (e.g., labels 5 (aminoglycoside), 7 (β-lactam), and 11 (rifampin)). However, prediction accuracy remains suboptimal for smaller datasets, particularly glycopeptide (label 9), chloramphenicol (label 10), and polymyxin (label 14) resistance categories, indicating areas for future improvement.

## 4 Discussion

Conventional AST, as the gold standard for detection, yields results that are susceptible to testing procedural variations and requires specific operational expertise and laboratory conditions. Moreover, it can only test one resistance phenotype at a time, requiring several days to complete (Govender et al., 2021). At the genetic level, techniques such as DNA microarrays, polymerase chain reaction (PCR), and quantitative PCR (qPCR) were previously employed to detect antibiotic resistance genes (ARGs) (Singh and Sodhi, 2024). The scarcity of primers is a major drawback of amplification-based techniques (Ovchinnikov et al., 2017).

In comparison, whole-genome/metagenome-based computational approaches are unaffected by issues of operational experience, laboratory environment, or primer scarcity, and can simultaneously detect multiple resistance phenotypes within minutes. Early computational methods primarily relied on sequence alignment and gene annotation (Zhou et al., 2020), exemplified by tools such as MG-RAST, AMR-Finder, and PATRIC. However, the lack of allelic variant specificity significantly impacts results because different variations confer distinct phenotypic resistance profiles (Liang et al., 2023). Furthermore, the difficulty in standardizing alignment parameters (e.g., similarity thresholds, coverage criteria) across different resistance genes frequently leads to elevated rates of both false-negative and false-positive predictions (Arango-Argoty et al., 2018). Artificial intelligence-based prediction of resistance genes and phenotypes addresses these limitations by learning intrinsic feature correlations from existing large-scale genomic sequences and resistance data, thereby effectively reducing both false-negative and false-positive prediction rates (Singh and Sodhi, 2024).

Building upon prior work utilizing nucleotide-level sequences, our study proposes a novel approach employing protein sequences and recently pre-trained protein language models for antimicrobial resistance (AMR) prediction. Compared to nucleotide-based methods, amino acid sequences offer three key advantages: (i) Enhanced functional specificity through direct capture of critical protein features (e.g., drug binding sites and efflux pump active-site variants) and precise identification of resistance-associated domains via conserved motif analysis (Tondnevis et al., 2020); (ii) Improved cross-species generalizability by eliminating host GC content bias (Zhang et al., 2025); and (iii) Superior computational efficiency, as the 20-letter amino acid alphabet reduces dimensionality vs. the 64 possible codon combinations, and at the same time, enabling effective transfer learning from protein language models. Our comparative results (Figure 8) demonstrate significant accuracy improvements in resistance gene prediction, while simultaneously providing novel insights into the essential characteristics of genetic material and proteins from a biolinguistics perspective.

However, the transition from gene to protein sequences for antibiotic resistance prediction may introduce prediction errors due to the loss of critical genomic information: (i) Synonymous mutations: while preserving amino acid sequences, these mutations can alter mRNA secondary structures (e.g., ribosome binding site stability) or introduce rare codons affecting translation rates, thereby modulating resistance gene expression levels (Wong et al., 2022); (ii) Non-coding functional elements: key regulatory features in promoters or untranslated regions (UTRs) that control gene expression are absent in protein sequences (Zrimec et al., 2021); (iii) Mobile genetic elements: resistance-associated markers from insertion sequences (IS) or transposase genes are not captured (Partridge et al., 2018). Although the loss of these critical genomic features has relatively minor impacts on resistance gene prediction, it substantially compromises the accuracy of bacterial phenotype prediction, which likely accounts for the observed discrepancies in our phenotypic resistance predictions (Figure 9).

Focusing on the machine learning model, its quality relies heavily on the feature extraction phase, which converts diverse data forms such as images, text, data, and sequences into machine-readable encoding while retaining the original data features and minimizing irrelevant noise (Yan et al., 2020). This study employs pre-trained protein language models ProtBert-BFD and ESM-1b, which not only address the issue of insufficient data for training feature extraction models from scratch but also leverage these pre-trained models to extract amino acid interactions and protein structural features from different perspectives, providing accurate, noise-reduced encoding for subsequent classification processes (Elnaggar et al., 2021; Rives et al., 2021). Compared to using a single data source and model, this approach captures more effective information, reduces data redundancy, and ultimately enhances predictive performance (Figure 7).

The classification model is the core architecture of the system. It is crucial to design an appropriate architecture and depth so that the model's parameter scale aligns with the training data size, allowing for precise extraction of useful information while avoiding noise (Garg et al., 2021). Through experiments with small-scale data, we found that a relatively simple three-layer LSTM architecture is better suited for lower-dimensional data (ProtBert-BFD encoding), whereas a more complex six-layer MH-LSTM architecture is better for higher-dimensional data (ESM-1b encoding). The primary reason might be that lower-dimensional data distributions are not complex, so deeper networks or additional MH structures may abstract features too much, leading to the loss of critical information and decreased model generalization performance (Atienza, 2022; Deng et al., 2022). On the other hand, deeper MH-LSTM architectures can alleviate the issue of parameter explosion with high-dimensional data and make the model focus more on the effective information in the hidden layers, reducing noise influence (Vaswani et al., 2017).

The experimental results demonstrate that our model significantly outperforms sequence alignment and conventional AI algorithms in reducing both false-negative and false-positive predictions of ARGs (Figure 8). Concurrently, it achieves notable reductions in false-positive rates for resistance phenotype predictions while improving accuracy for specific phenotypes (Figure 9). High-accuracy ARG prediction enables AI to (i) Detect novel/rare ARG variants (Sodhi and Singh, 2022); (ii) Elucidate evolutionary pathways, e.g., horizontal gene transfer, mutation accumulation (Singh and Sodhi, 2024); (iii) Identify previously undetected ARGs beyond conventional methods' detection limits (Sodhi et al., 2023). For instance, AI-based model could predict distant ARG variants (< 30% homology to known genes) revealing novel resistance protein families and “silent” chromosomal resistance clusters (e.g., stress-inducible antibiotic-inactivating enzymes) (Singh et al., 2024).

Current AI-driven phenotype prediction holds transformative potential by potentially obviating laboratory culturing in infection diagnostics. However, pending resolution of implementation challenges, research focus remains on its theoretical promise rather than demonstrated clinical workflow impacts (d'Humières et al., 2021). Our model's high-precision phenotype prediction and low false-positive rates can guide targeted antibiotic use, reducing unnecessary broad-spectrum antibiotic reliance. In urinary tract infections (UTIs)—where rising antimicrobial resistance forces increasing broad-spectrum use—AI-based model could optimize empirical prescribing through rapid susceptibility profiling (Kanjilal et al., 2020). This enables tailored antibiotic selection for uncomplicated UTIs within 2 h vs. 48–72 h for conventional AST.

Overall, with the continuous expansion of subsequent datasets and ongoing optimization of algorithmic models, AI models are expected to progressively enhance their practical guidance significance for clinical treatment.

## Data Availability

All data associated with this study have been deposited in a publicly available repository to help other researchers evaluate our findings and build on our work. The codes and data used in this study are available on GitHub https://github.com/wr-sky/ARGs.
