# Supplementary material for: Predicting antibiotic resistance genes and bacterial phenotypes based on protein language models
Source: Front Microbiol. 2025 Sep 8;16:1628952. doi: 10.3389/fmicb.2025.1628952 (PMC12450889; doi:10.3389/fmicb.2025.1628952)
Supplement: Supplementary file 1 [file Table_1.docx]

**Supplementary File 1. Common Methods for Protein Sequence Feature Extraction.**

| Embedding | Model | Feature | | | | | |
| --- | --- | --- | --- | --- | --- | --- | --- |
|  |  | Model features | | | | | Technical feature |
|  |  | Deep learning technology | Database | Layers | Parameters | Dimension |  |
| One-hot | - | - | | | | | Intuitive representation of amino acids, sparse data with no contextual relationships. |
| PSSM | - | - | | | | | Dependent on specific structures, lacking global information. |
| ESM | ESM-2 ^1^ | Transformer | UniRef50 | 6-48 | 8M-15B | 320-5120 | Predicting structure, function, and other protein properties from a single protein sequence. |
|  | ESMFold ^1^ | BERT | PDB+  UniRef50 | 48 (+36) | 690M (+3B) | - | End-to-end 3D structure prediction from a single protein sequence. |
|  | ESM-1b ^2^ | Transformer | UniRef50 | 33 | 650M | 1280 | Extracting protein embedding vectors. |
|  | ESM-1v ^3^ | Transformer | UniRef90 | 33 | 650M | 1280 | Used for predicting the effects of mutations, i.e., predicting the functional impact of sequence changes. |
|  | ESM-IF1^4^ | Transformer | CATH +UniRef50 | 20 | 124M | 512 | Inverse folding models used to design sequences for a given structure and predict the impact of sequence variations on function. |
| ProtTrans ^5^ | ProtTXL | Transformer Decoder | BFD100 | 32 | 562M | - | Extracting protein embedding vectors. |
|  |  |  | UniRef100 | 30 | 409M |  |  |
|  | ProtBert | BERT | BFD100 | 30 | 420M | 30 | Extracting protein embedding vectors. |
|  |  |  | UniRef100 |  |  |  |  |
|  | ProtXLNet | Transformer Decoder | UniRef100 | 30 | 409M | 1024 | Using a memory-like mechanism to handle sequences of arbitrary length, allowing bidirectional context to be captured within a single memory segment. |
|  | ProtAIbert | Albert | UniRef100 | 12 | 224M | 4096 | Extracting protein embedding vectors. |
|  | ProtElectra | Electra | UniRef100 | 30 | 420M | 1280 | Sequence generation model |
|  | ProtT5-XL | Transformer | BFD100 | 24 | 3B | 1024 | Predicting from a single protein sequence, while also being capable of making predictions beyond the actual length range of positional encoding. |
|  |  |  | UniRef50 |  |  |  |  |
|  | ProtT5-XXL | Transformer | BFD100 | 24 | 11B | 1024 | Predicting structure and function from a single protein sequence. |

1 Lin, Z. *et al.* Evolutionary-scale prediction of atomic-level protein structure with a language model. *Science* **379**, 1123-1130 (2023).

2 Rao, R., Meier, J., Sercu, T., Ovchinnikov, S. & Rives, A. Transformer protein language models are unsupervised structure learners. *Biorxiv*, 2020.2012. 2015.422761 (2020).

3 Meier, J. *et al.* Language models enable zero-shot prediction of the effects of mutations on protein function. *Advances in neural information processing systems* **34**, 29287-29303 (2021).

4 Hsu, C. *et al.* Learning inverse folding from millions of predicted structures. in *International conference on machine learning.* 8946-8970 (2022).

5 Elnaggar, A. *et al.* Prottrans: Toward understanding the language of life through self-supervised learning. *IEEE transactions on pattern analysis and machine intelligence* **44**, 7112-7127 (2021).
